# Nitric Oxide and Reactive Oxygen Species Coordinately Regulate the Germination of *Puccinia striiformis* f. sp. *tritici* Urediniospores

**DOI:** 10.3389/fmicb.2016.00178

**Published:** 2016-02-23

**Authors:** Shuining Yin, Zhijuan Gao, Chenfang Wang, Lili Huang, Zhensheng Kang, Hongchang Zhang

**Affiliations:** ^1^State Key Laboratory of Crop Stress Biology for Arid AreasYangling, China; ^2^College of Life Sciences, Northwest A&F UniversityYangling, China; ^3^College of Forestry, Northwest A&F UniversityYangling, China; ^4^College of Plant Protection, Northwest A&F UniversityYangling, China

**Keywords:** NO, ROS, *Pst*

## Abstract

Nitric oxide (NO) and reactive oxygen species (ROS) function as signaling molecules in a number of critical signal transduction pathways in plants, including plant biotic interactions. In addition to the role of plant-derived NO and ROS in plant resistance, which has been well documented, pathogen-produced NO and ROS have recently emerged as important players in fungal development and pathogenesis. However, the effects of pathogenic fungi-derived NO and ROS on signaling pathways during fungal pre-infection development remain unknown. Here, using a combination of pharmacological approaches and confocal microscopy, we investigated the roles of NO and ROS during the germination of *Puccinia striiformis Westend* f. sp. *tritici* (*Pst*) the wheat stripe rust pathogen. Both NO and ROS have a crucial role in uredinial germination. The scavengers of NO and ROS delayed spore germination and decreased the lengths of germ tubes. A similar phenotype was produced after treatment with the promoter. However, the spores germinated and grew normally when the levels of NO and ROS were simultaneously elevated by the application of a promoter of NO and a donor of ROS. Confocal laser microscopy indicated that both NO and ROS preferentially localized at the germ pores and apexes of growing germ tubes when the ROS/NO ratio in the spores was maintained in a specific range. We concluded that both NO and ROS are critical signaling molecules in the pre-infection development of *Pst* and that the polar growth of the germ tube is coordinately regulated by NO and ROS.

## Introduction

Two important types of free radicals, NO and ROS, are crucial signaling molecules involved in a number of signal transduction pathways. The roles of NO and ROS in mammals have been studied for many years. They are crucial messengers in the immune, nervous, and cardiovascular systems (Palmer et al., 1987). In plants, they are involved in several physiological processes, including seed germination and lateral leaf and root development, and have been implicated in both abiotic and biotic stress responses (Besson-Bard et al., 2008; Wilson et al., 2008; Swanson and Gilroy, 2010). Indeed, there is considerable evidence that plant-derived NO and ROS are important in initiating plant responses to pathogens or elicitors (Brisson et al., 1994; Levine et al., 1994; Delledonne et al., 1998; Chaki et al., 2009).

Evidence is also emerging that NO and ROS are important regulatory molecules in microbe, including plant pathogens. It has been found that ROS is involved in the germination and germ tube growth of conidia of *Cladosporium fulvum* (Lu and Higginsf, 1999) and the biofilm resistance of *Pseudomonas aeruginosa* (Elkins et al., 1999).

It was reported that pathogen-derived NO influences germination in *Colletotrichum coccodes* (Wang and Higgins, 2005), conidiation in *Coniothyrium minitans* (Gong et al., 2007) and sporangiophore development in *Phycomyces blakesleeanus* (Maier et al., 2001) and affects the formation of appressoria in the obligate biotrophic powdery mildew fungus *Blumeria graminis* (Prats et al., 2008) and pathogenicity in the rice blast fungus *Magnaporthe oryzae* (Averyanov and Lapikova, 1990).

Reactive oxygen species have been reported to be involved in fungal virulence and development (Heller and Tudzynski, 2011). There are various reports on the effects of ROS free radicals on spore germination. O_2_^-^ and OH radicals were both detected during the germination of *Pyricularia oryzae*, and the radical scavengers superoxide dismutase (SOD), catalase and OH increased the percentage of germination (Averyanov and Lapikova, 1990). During spore germination in *Neurospora crassa*, an accumulation of catalase was observed, indirectly suggesting that H_2_O_2_ was generated in the process (Michan et al., 2002).

Wheat stripe rust, caused by *Pst*, is one of the most important diseases of wheat and can cause significant loss to wheat yield and grain quality (Chen, 2005). Although *Pst* is a macrocyclic rust pathogen, its propagation and spread occur primarily by means of urediniospores, which are capable of germination and infection under suitable environmental conditions immediately after release (Chen et al., 2014). Following the initiation of germination, the cytoplasm of a urediniospore moves into the germ tube until it reaches a stoma.

To our knowledge, no study has suggested a role for NO and ROS during urediniospore germination. Hence, CLSM was used to visualize NO and ROS generated by urediniospores *in vivo* and the role of NO and ROS in *Pst* development was investigated. We identify a regulatory role for NO and ROS during the germination of urediniospores and the apical growth of germ tubes in *Pst* using specific probes and the donor/promoter and scavengers of NO and ROS. The scavengers of NO and ROS delayed germination and decreased germ tube length. Moreover, a promoter of NO and a donor of ROS could also delay germination and decrease the germ tube length. Further study determined that the spores germinate when the ROS/NO ratio is maintained within a specific range and that NO and ROS primarily exist in the apex of the germ tube, suggesting that both NO and ROS are involved in apical germ tube growth.

## Materials and Methods

### Pathogen and Reagents

Fresh urediniospores of *Pst* pathotype CYR31 used in this study were provided by the Institute of Plant Pathology, Northwest A&F University. The specific NO scavenger c-PTIO (Balcerczyk et al., 2005), the substrate of NO synthesis L-Arg (Bonilla et al., 2004; for clarity, L-Arg is elsewhere called “the promoter of NO”), the ROS donor triphosphopyridine nucleotide (NADPH), the NADPH scavenger DPI, NO-specific probe 4-amino-5-(*N*-methylamino)-2,7-difluorofluorescein diacetate (DAF-FM DA) and the ROS-specific probe 2′,7′-dichlorodihydrofluorescein diacetate (H_2_DCF DA) were used in this study. All reagents used in this study were obtained from Sigma–Aldrich, USA.

### Determination of Appropriate Reagent Concentrations

Different reagents at different concentrations (c-PTIO at 0, 50, 100, 150 μM; L-Arg at 0, 2, 3, 4 mM; DPI at 0, 10, 20, 30 μM and NADPH at 0, 2.5, 3.5, 4.5 mM) were tested in order to find the appropriate concentrations for use in the experiments.

Different probes at different concentrations (DAF-FM DA at 1, 2, 5, 10, and 15 μM and H_2_DCF DA at 20, 30, 50, 70, and 100 mM) were tested in order to find the appropriate concentrations to detect the generation of NO and ROS in the study.

Finally, concentration of c-PTIO at 100 μM, L-Arg at 2 mM, DPI at 20 μM, NADPH at 2.5 mM, DAF-FM DA at 10 μM and H_2_DCF DA at 50 mM were selected.

### Effects of NO and ROS on Urediniospore Germination and Germ Tube Growth

Fresh urediniospores (0.6 mg) were added to 10 ml distilled water, 10 ml 100 μM c-PTIO, 10 ml 2 mM L-Arg, 10 ml 20 μM DPI, 10 ml 2.5 mM NADPH, 5 ml 20 μM DPI+5 ml 100 μM c-PTIO (c-PTIO +DPI), 5 ml 2 mM L-Arg+5 ml 2.5 mM NADPH (L-Arg +NADPH), 5 ml 20 μM DPI+5 ml 2 mM L-Arg (L-Arg +DPI) or 5 ml 2.5 mM NADPH+5 ml 100 μM c-PTIO (c-PTIO +NADPH) and allowed to germinate at 9°C in darkness.

### Evidence for, and Localization of, Endogenous NO and ROS in Germinating *Pst* Urediniospores

The specific fluorescence probes-H_2_DCF DA and DAF-FM DA were used to detect ROS and NO. In the presence of ROS and NO, H_2_DCF DA and DAF-FM DA were converted to fluorescent DCF and DAF-FM triazole (DAF-FM T), which could be detected separately by bright green fluorescence in CLSM.

Germinating urediniospores treated as described above (see Effects of NO and ROS on Urediniospore Germination and Germ Tube Growth) were collected at different time points and mixed with 10 μM DAF-FM DA or 50 mM H_2_DCF DA prior to incubation in darkness at 25°C for 30 min. They were then immersed twice for 10 min in Tris-HCl buffer (50 mM, pH 7.4) to remove excess dye. A distilled water control without DAF-FM DA or H_2_DCF DA staining and with the same other procedures was treated as CK to detect the autofluorescence of germinating urediniospores. Then, the urediniospores were placed on slides to detect the generation of NO and ROS by CLSM (LSM 510 META, Zeiss Corporation, Germany). Fluorescence was detected at an excitation frequency of 488 nm, and emission was filtered using a 515–530 nm barrier filter. NO and ROS were detected with the same parameters (gain, magnification and so on) across all treatments. Images were recorded after DAF-FM DA and H_2_DCF DA staining, and observations of the CK enabled discrimination between autofluorescence (also excited by the argon laser) and fluorescence due to NO and ROS generation. The MFI values were measured in different locations of the urediniospores and germ tubes using Image Pro Plus software (IPP software, USA).

### Rates of Germination and Germ Tube Lengths

Germinating urediniospores were collected at different time points and placed on slides to count the numbers of germinated urediniospores and to measure the lengths of germ tubes (a germ tube length greater than one-half the spore diameter was defined as germination) using an Olympus BX51 microscope (Olympus Corporation, Japan). The germination rate was expressed as a percentage based on 100 urediniospores.

### Statistical Analysis

One hundred urediniospores were analyzed in every treatment at random, and all experiments were performed at least three times. Only representative images are shown in the paper. Differences in germination rates, germ tube lengths and mean pixel intensity among the treatments were analyzed by one-way ANOVA with the least significant differences (LSD) test at 0.05 probability level. All statistical tests were performed using SPSS 16.0 (SPSS Inc., Chicago, IL, USA).

## Results

### Promoter and Scavenger of NO and ROS Affect Spore Germination

**FIGURE 1:**
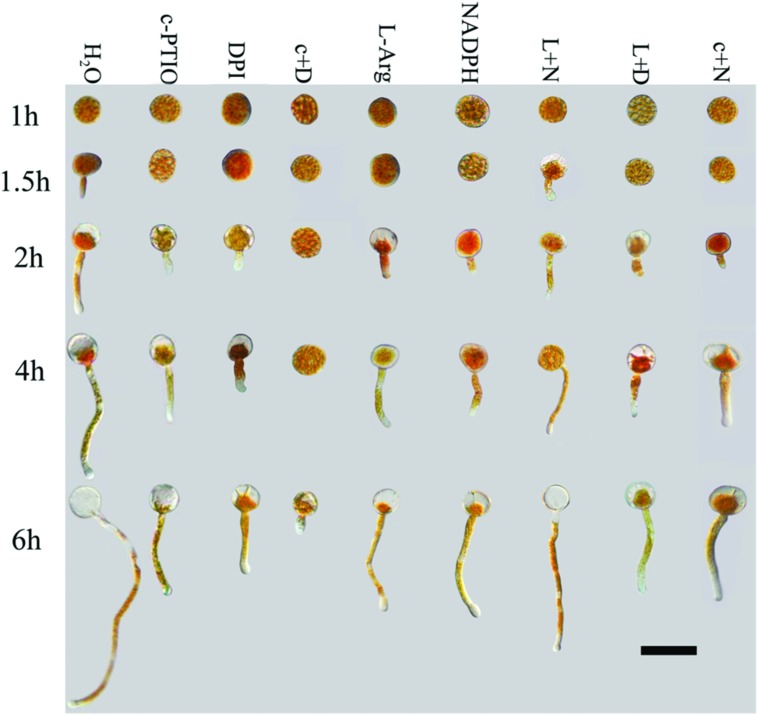
**Growth of *Pst* urediniospores after different treatments and time points.** Deficiencies of ROS or NO inhibited germination of urediniospores and germ tube growth. Increases in ROS alone or NO alone did not promote germination or germ tube growth. Only increases in both ROS and NO led to increased spore germination. C+D, c-PTIO +DPI; L +N, L-Arg +NADPH; L+D, L-Arg +DPI; c+N, c-PTIO +NADPH. Scale bar, 50 μm.

**FIGURE 2:**
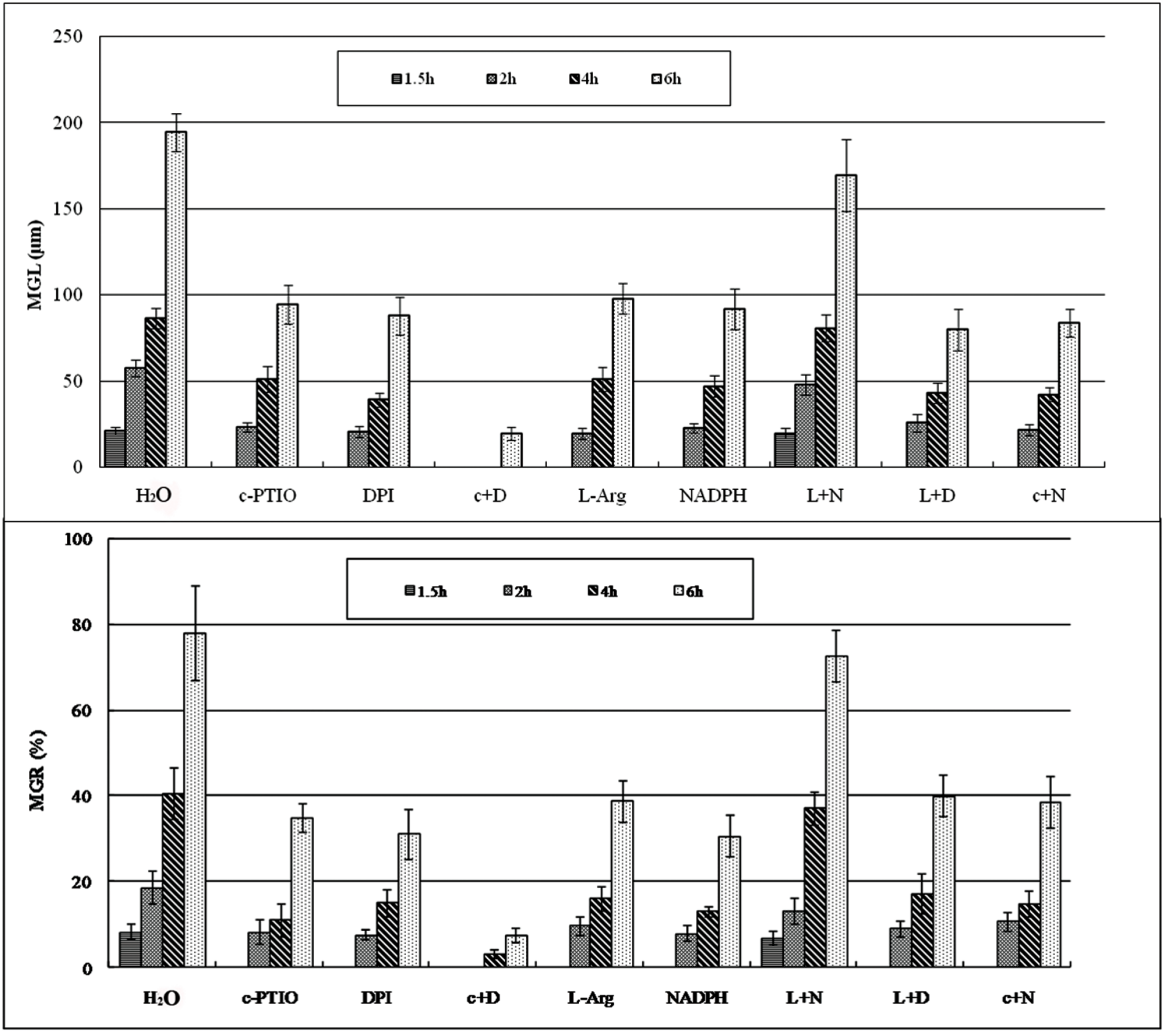
**The mean germ tube lengths and mean germination rate during *Pst* spore germination after different treatments at different time points.** MGL, mean germ tube length; MGR, mean germination rate.

After treatment with c-PTIO and DPI, spore germination was significantly suppressed and delayed in germination time compared with spores treated with distilled water (**Figures 1** and **2**), as measured by MGL and MGR.

Urediniospores began to germinate after 1.5 h in distilled water, and the MGR and MGL were 8% and 21.3 μm, respectively; they increased over time, especially at 4–6 hpg, and peaked at 6 hpg (78.2% and 194.6 μm). By contrast, the urediniospores maintained dormancy for 1–1.5 hpg when treated with DPI and c-PTIO at 2 hpg. The MGR values were 7.7 and 8.3%, and the MGL values were 20.8 and 23.4 μm, respectively. Although the MGR and MGL increased over time, there were significant differences compared with the distilled water control. Thus ROS and NO had important roles in the germination of spores and in germ tube growth.

After treatment with L-Arg and NADPH, urediniospore germination was also significantly suppressed (**Figures 1** and **2**). The data for MGR and MGL showed no differences between L-Arg, NADPH, c-PTIO, and DPI treatments at any time points, which suggested that an increase in ROS only or NO only could not promote spore germination or germ tube growth.

After treatment with L-Arg +DPI and c-PTIO +NADPH, the MGL and MGR were increased compared with the DPI and c-PTIO treatment (**Figures 1** and **2**). However, variance analysis showed no significant difference, indicating that excess NO or ROS did not promote spore germination or germ tube growth.

Germination was almost completely suppressed after treatment with c-PTIO +DPI (**Figures 1** and **2**), and variance analysis indicated significant differences compared with other treatments, especially distilled water.

In the presence of L-Arg +NADPH, spore germination was indistinguishable from that observed in distilled water (**Figures 1** and **2**). However, there was a significant difference between L-Arg +NADPH and distilled water at 4 hpg.

Thus, spore germination and fungal growth were significantly reduced when NO or ROS levels were decreased (**Figures 1** and **2**), and increases in ROS only or NO only failed to promote increases (**Figures 1** and **2**), suggesting that NO and ROS play a crucial role in spore germination and germ tube growth. This result suggested that an optimum ratio of ROS to NO possibly exist during urediniospore germination in *Pst*.

### The Fluorescence Intensity of Endogenous NO Generated During Urediniospore Germination Following Different Treatments

**FIGURE 3:**
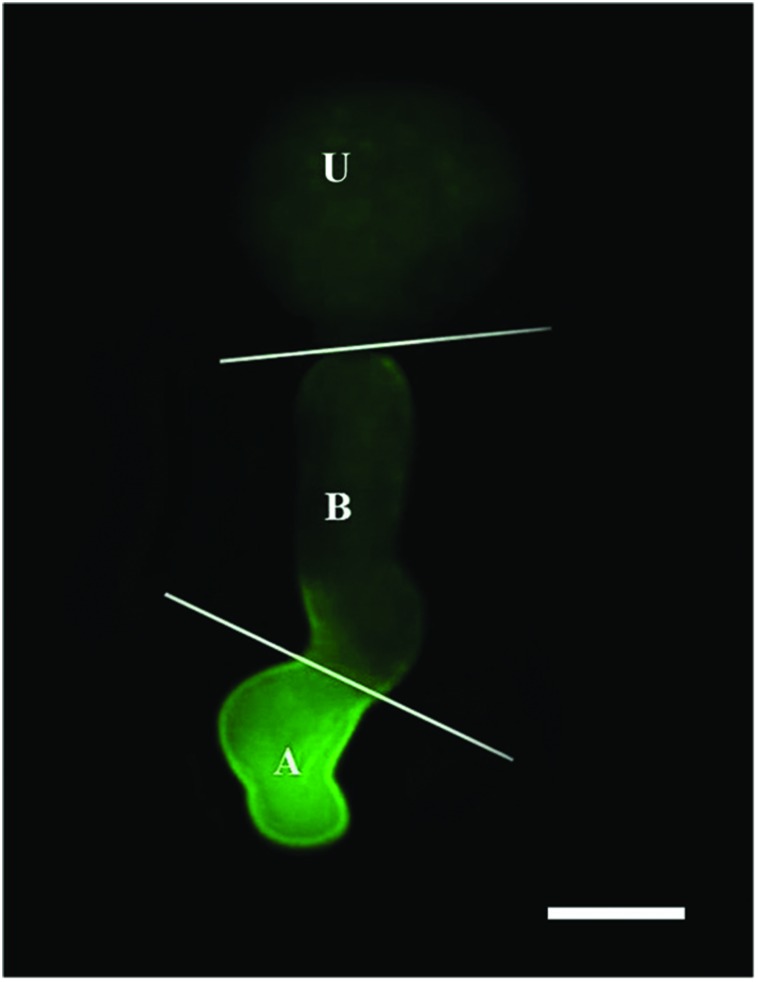
**Image of a germinating urediniospore at 2 hpg when stained with DAF-FM DA.** Intense green fluorescence indicates the presence of NO in the apex of the germ tube. U, urediniospore; B, base of germ tube; A, apex of germ tube. Scale bar, 10 μm.

Nitric oxide-specific fluorescent probe DAF-FM DA and CLSM were used to detect NO produced during urediniospore germination. The urediniospore and the germ tube were divided into three parts (urediniospore, base of germ tube and apex of germ tube), as shown in **Figure 3**.

CSLM performed after DAF-FM DA staining revealed the generation of NO in *Pst* spores and germ tubes.

**FIGURE 4:**
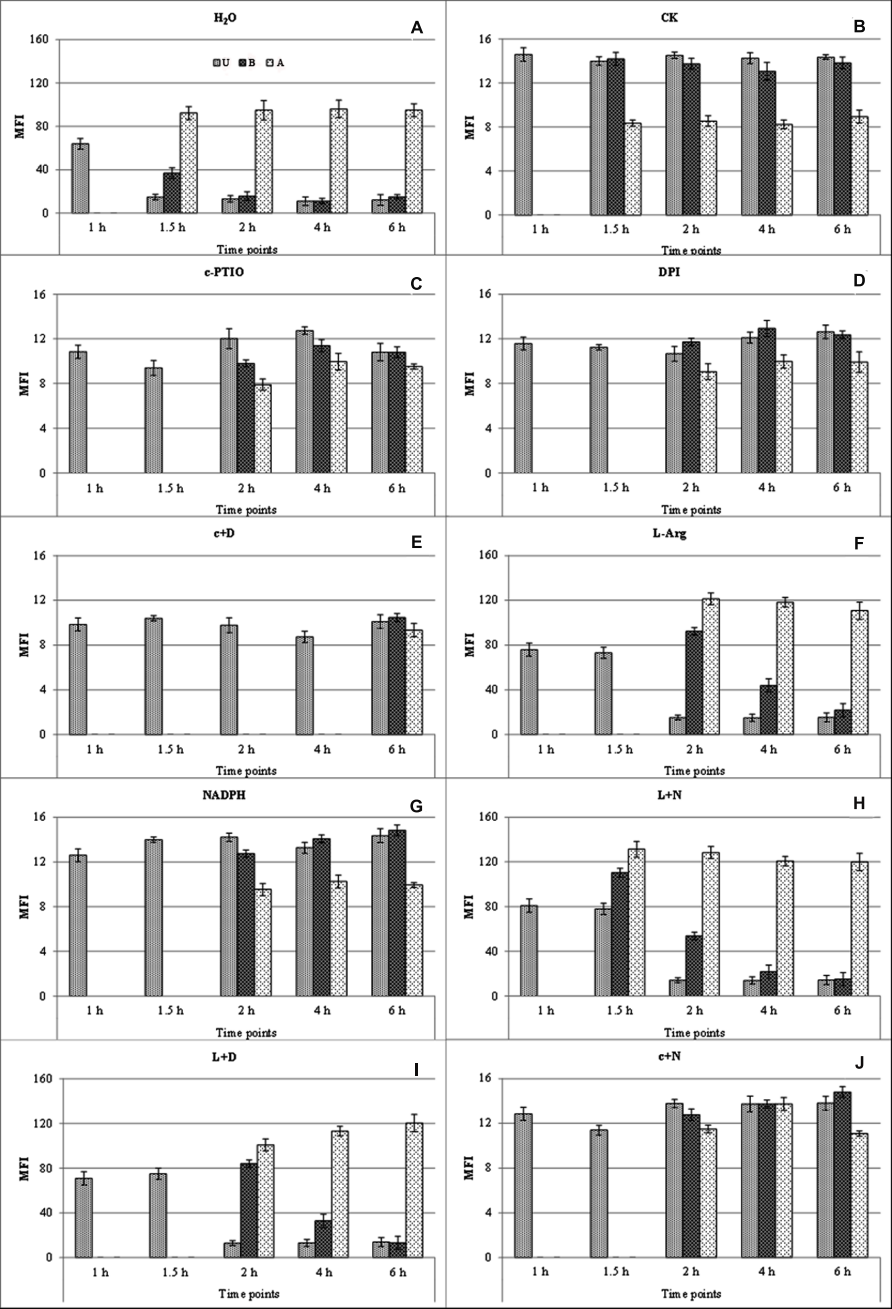
**Mean fluorescence intensity of endogenous NO generated during germination at different time points after different treatments.** Bright fluorescence was observed after treatment with distilled water (H_2_O) and DAF-FM DA **(A)**; faint fluorescence was observed in distilled water without DAF-FM DA staining (CK) **(B)**. U, urediniospore; B, base of germ tube; A, apex of germ tube. c+D, c-PTIO +DPI; L+N, L-Arg +NADPH; L+D, L-Arg +DPI; c+N, c-PTIO +NADPH.

Bright fluorescence was observed after treatment with distilled water and DAF-FM DA (**Figure 4A**); faint fluorescence was observed in distilled water without DAF-FM DA staining (CK; **Figure 4B**); c-PTIO-treated samples also displayed faint and uniform fluorescence throughout the study (**Figure 4C**). Combining the results of these three treatments, it could be confirmed that the fluorescence in **Figure 4B** is the autofluorescence of urediniospore, and the bright green fluorescence in **Figure 4A** is due to NO generation and not autofluorescence.

Bright green fluorescence was detected in germ pores, indicating that NO was generated around the germ pores during the urediniospore water-swelling stage at 1 hpg. However, germ tubes emerged from the germ pores at 1.5–2 hpg, and staining showed pronounced fluorescence in most short germ tubes, especially at their tips (**Figure 4A**). Further images showed intense fluorescence localized at the apices of germ tubes at 2–6 hpg, although faint fluorescence was also observed in the spores and bases of the germ tubes (**Figure 4A**). The same trends were observed after treatment with L-Arg +NADPH (**Figure 4H**), L-Arg (**Figure 4F**), and L-Arg +DPI (**Figure 4I**), although germination was delayed in the last two treatments.

After treatment with DPI or NADPH, visual inspection and staining with DAF-FM DA showed a constant faint fluorescence in the spores and germ tubes throughout the study (**Figures 4D,G**), showing that both the suppression and promotion of ROS inhibited NO.

When ROS and NO were both inhibited, the MFI of NO was decreased (**Figure 4E**), and spore germination was considerably delayed (**Figures 1** and **2**). After treatment with c-PTIO +NADPH, staining with DAF-FM DA showed faint fluorescence in the spores and germ tubes (**Figure 4J**).

Urediniospores treated with L-Arg, L-Arg +NADPH and L-Arg +DPI, when stained with DAF-FM DA, displayed a marked bright fluorescence correlated with the development of germ tubes (**Figures 4F,H,I**).

After treatment with L-Arg +DPI, the bright fluorescence at the spores and germ tubes were observed (**Figure 4I**), which suggested that although L-Arg could help to relieve ROS depression and produce more NO, it exhibited no enhancement on the elongation of germ tubes (**Figures 1** and **2**).

It was also observed that only when the ROS and NO were simultaneously added (**Figure 4H**) could the spore germinate normally, as in the distilled water treatment (**Figures 1** and **2**).

These results show that NO plays a crucial role in apical growth and that ROS is involved in the generation of NO. NO production was greatly increased by L-Arg, further suggesting a role for a NOS enzyme as the source of NO generation in the fungus.

### Fluorescence Intensity of Endogenous ROS Generated During Germination After Different Treatments

Reactive oxygen species-specific fluorescent probe H_2_DCF DA and CLSM were used to detect the ROS produced during urediniospore germination.

**FIGURE 5:**
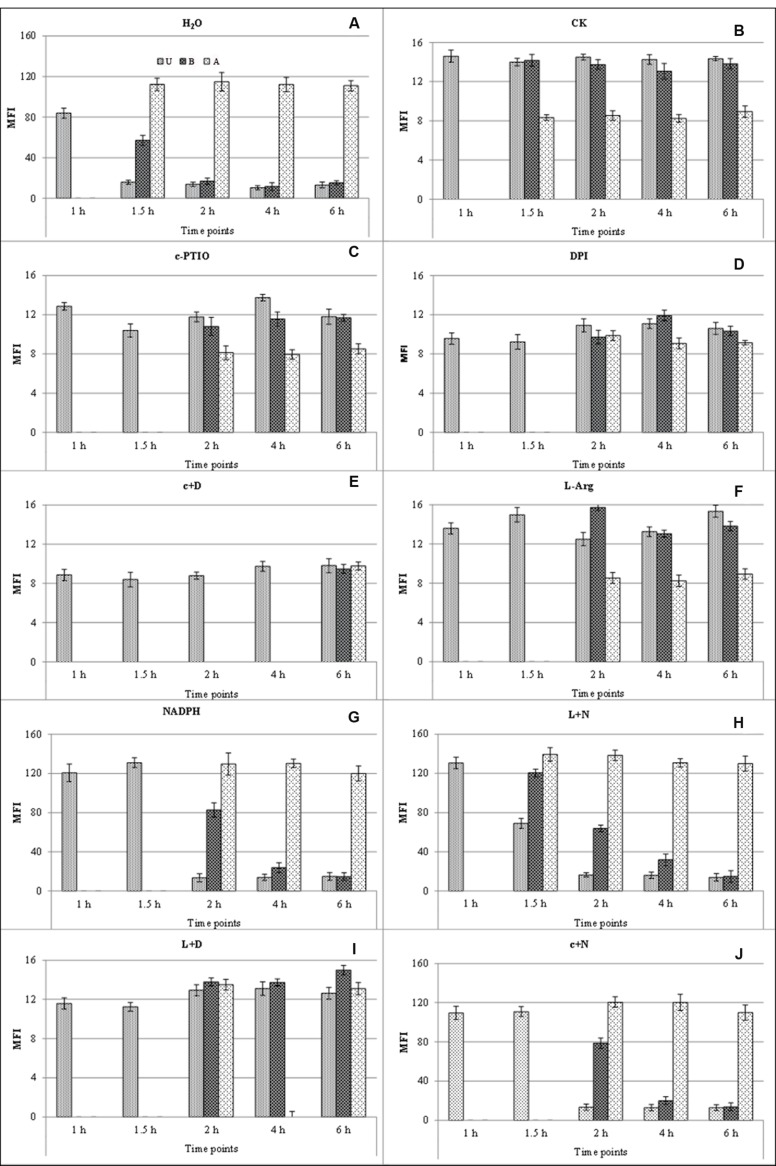
**Mean fluorescence intensity of endogenous ROS generated during germination at different time points and treatments.** Bright fluorescence was observed after treatment with distilled water (H_2_O) and H_2_DCF DA **(A)**; faint fluorescence was observed in distilled water without H_2_DCF DA staining (CK) **(B)**. U, urediniospore; B, base of germ tube; A, apex of germ tube. c+D, c-PTIO +DPI; L+N, L-Arg +NADPH; L+D, L-Arg +DPI; c+N, c-PTIO +NADPH.

Bright green fluorescence was observed for the treatment with distilled water and H_2_DCF DA (**Figure 5A**); faint fluorescence was observed in the distilled water without H_2_DCF DA staining (CK; **Figure 5B**); DPI-treated samples also displayed faint and uniform fluorescence throughout the study (**Figure 5D**). Combining the results of these three treatments, it could be confirmed that the fluorescence in **Figure 5B** was the autofluorescence of urediniospores, whereas the bright green fluorescence in **Figure 5A** was due to ROS generation and not autofluorescence.

Bright green fluorescence indicated that ROS was generated around germination pores during the water-swelling stage at 1 hpg (**Figure 5A**). During the growth of the germ tubes, the characteristic of ROS generation were similar to DAF-FM DA staining (**Figure 5A**). The same trends were observed after treatment with L-Arg +NADPH (**Figure 5H**), NADPH (**Figure 5G**) and c-PTIO +NADPH (**Figure 5J**), although the germination was delayed in the last two treatments.

After treatment with the NO scavenger c-PTIO or the NO promoter L-Arg and staining with H_2_DCF DA, only faint fluorescence was detected at the tips of germ tubes (**Figures 5C,F**). This result indicated that either the suppression or the promotion of NO decreased the concentration of ROS during spore germination.

Urediniospore treatment with NADPH, L-Arg +NADPH or c-PTIO +NADPH and staining with H_2_DCF DA showed a markedly bright fluorescence during the germination of urediniospores (**Figures 5G,H,J**).

When the ROS and NO were both restrained, the MFI of ROS was decreased (**Figure 5E**) and spore germination was delayed (**Figures 1** and **2**) which was similar to DAF-FM DA staining.

After treatment with L-Arg +DPI and staining with H_2_DCF DA, the urediniospore showed faint fluorescence in the spores and germ tubes (**Figure 5I**).

There was a significant difference between treatments with c-PTIO and c-PTIO +NADPH in the urediniospores at 1 hpg and in the apex of germ tubes at 2–6 hpg after H_2_DCF DA staining (**Figures 5C,J**). However, the MGL were not increased (**Figures 1** and **2**), which suggested that although NADPH could help to relieve NO depression and produce more ROS, it did not enhance the elongation of germ tubes.

It was also observed that only when the ROS and NO were promoted simultaneously (**Figure 5H**), the spore could germinate normally as in the distilled water treatment (**Figures 1** and **2**).

These results show that ROS plays a crucial role in the apical growth of urediniospores and NO is involved in the generation of ROS. ROS production was greatly increased by NADPH, further suggesting a role for NADPH as the source of ROS generation in the fungus.

### ROS/NO Ratio

The fluorescence intensity was directly proportional to the content of NO or ROS, so the ratio of ROS/NO fluorescence intensity could indirectly reflect the ratio of ROS/NO content in germinated urediniospores.

The MFI values of urediniospores treatment with H_2_O and L-Arg +NADPH (the urediniospores after the two treatments could germinate normally) were recorded and analyzed (**Table 1**).

**Table 1:** Reactive oxygen species/NO ratio after H_2_O and L+N treatments.

Treatment	Localization	Free radicals	Time points				
			1 h	1.5 h	2 h	4 h	6 h
H_2_O	U	ROS	83.8 ± 5.3	15.7 ± 2.1	13.9 ± 1.5	10.3 ± 2.7	13.0 ± 3.1
		NO	63.8 ± 4.1	14.7 ± 1.3	13.1 ± 1.3	11.0 ± 2.7	12.1 ± 3.0
		ROS/NO	1.3	1.1	1.1	0.9	1.1
	B	ROS	0	57.0 ± 5.1	16.8 ± 2.7	11.6 ± 3.0	15.3 ± 2.1
		NO	0	37.0 ± 5.2	15.7 ± 2.2	11.2 ± 1.4	15.0 ± 2.8
		ROS/NO	/	1.5	1.1	1.0	1.0
	A	ROS	0	112.2 ± 7.1	114.8 ± 8.4	112.0 ± 7.0	110.9 ± 7.1
		NO	0	92.2 ± 6.0	94.8 ± 8.4	96.0 ± 8.1	94.9 ± 6.2
		ROS/NO	/	1.2	1.2	1.2	1.2
L+N	U	ROS	130.9 ± 5.9	68.7 ± 5.0	16.5 ± 2.2	15.5 ± 3.2	14.0 ± 3.0
		NO	80.9 ± 5.9	78.0 ± 6.7	14.2 ± 1.9	14.0 ± 3.7	14.4 ± 4.0
		ROS/NO	1.6	0.9	1.2	1.4	1.0
	B	ROS	0	120.4 ± 5.2	63.9 ± 5.0	31.9 ± 3.2	14.9 ± 2.2
		NO	0	110.4 ± 4.4	53.9 ± 3.4	21.9 ± 5.9	15.2 ± 5.9
		ROS/NO	/	1.1	1.2	1.5	1.0
	A	ROS	0	139.3 ± 8.2	138.3 ± 9.5	130.7 ± 8.2	129.9 ± 9.2
		NO	0	131.3 ± 7.4	128.3 ± 5.3	120.7 ± 4.3	119.9 ± 7.7
		ROS/NO	/	1.1	1.1	1.1	1.1

*The MFI values of ROS and NO of the spores treated with H_2_O and L + N (spores after the two treatments could germinate normally) was collected. The ratio of ROS/NO was calculated at different locations at different time points. U, urediniospore; B, base of germ tube; A, apex of germ tube.*

The ratio of ROS/NO at different locations and time points showed that spores germinated normally when the ROS/NO ratio maintained within the range of 0.9–1.6.

## Discussion

In eukaryotes, NO is generated from many oxynitrides such as nitrite (NO_2_^-^) under acid conditions (Castello et al., 2008). In addition to chemical synthesis, NO can be generated by enzymatic reactions. In animals, NO is synthesized from O_2_ and L-Arg by different NO synthase (Bonilla et al., 2004) isoforms (Nowles and Moncada, 1994). Ninnemann and Maier (1996) reported NO synthase activities in fungi for the first time. NO synthase activity was detected during sporulation in *Blastocladiella emersonii* (Bonilla et al., 2004), and activity decreased significantly with the addition of L-NAME (Vieira et al., 2009). These results suggested that there was an enzymatic pathway of NO synthesis in fungi that was similar to that of mammals. In our study, endogenous NO increased sharply when the substrate of NO synthase L-Arg was added. L-Arg generates NO through the NOS catalysis pathway. Green fluorescence could still be observed after staining with the specific NO fluorescence probe, and the intensity fluorescence decreased dramatically after c-PTIO treatment. Therefore, endogenous NO might be generated by the NOS pathway during the germination of *Pst* urediniospores. It has been suggested that NO synthesis in phytopathogenic fungi is derived from an L-Arg-dependent pathway by a NOS-like system, as in the ascomycete fungi *C. coccodes* and *B. graminis* (Wang and Higgins, 2005; Prats et al., 2008). However, fungi do not contain NOS-like sequences in their genomes, except for *Aspergillus* species and *Glomerella graminicola* (Turrion-Gomez and Benito, 2011). Genetic studies indicated that NO synthesis in *M. oryzae* was not associated with an arginine-dependent pathway, although relatively weak NOS-like sequences were present in the genome (Samalova et al., 2013). It should be feasible to validate this result by identifying the NOS in *Pst* using the available genome sequence (Zheng et al., 2013).

There is growing evidence that certain specific enzymes, such as NADPH oxidase (NOX), produce ROS to regulate cellular functions, such as immunity, cell proliferation, cell differentiation, signal transduction, and ion transport (Finkel, 2003; Foreman et al., 2003; Kwak et al., 2003; Lambeth, 2004). In this study, it was found that although spore germination was restrained after NADPH treatment, a mass of green fluorescence was still detected by the ROS-specific fluorescence probe, and the intensity of this green fluorescence decreased dramatically after DPI treatment. Thus, during urediniospore germination, endogenous ROS might be generated by the NADPH pathway.

During the study, we observe that the wax layer and the epidermal hairs on the surface of the wheat leaves would cause the gathering of ROS/NO fluorescence probes which could interfere with the accuracy of the results by CLSM. This causes the difficulty to the research of the role of NO and ROS during the germination of urediniospore. Fortunately, *Pst*, as an obligate biotroph urediniospores, can germinate and form a germ tube on the water surface which cause the feasibility of the research in the pre-infection process *in vitro* under controlled conditions.

After hydration, a urediniospore germinates and develops a germ tube that can extend along the water surface. During germination, it is believed that fungal spores undergo an initial period of isotropic expansion associated with the uptake of water. Upon the establishment of a polarity axis, a short germ tube emerges and grows by apical extension, which is a defining feature of the filamentous fungi (Harris, 2006; Riquelme, 2013). Thus, the pre-infection development of *Pst* essentially involves the transition from isotropic growth to polarized growth, and it has been proposed that polarized hyphal growth requires the establishment of polarity during spore germination and maintenance of polarity during germ tube elongation (Momany, 2002). In this study, NO and ROS preferentially localized to the spore pore and apical region of the germ tube, suggesting that they are associated with these processes. Accumulating evidence indicates that there is a correlation between ROS production by NADPH oxidase and the polarized growth of fungal cells (Glasauer and Chandel, 2013). Localized production of ROS at the growing hyphal tips was detected by NBT or H_2_DCF DA staining for several fungal species, including *Epichlöe festucae*, *M. grisea*, and *Aspergillus nidulans* (Tanaka et al., 2006; Egan et al., 2007; Semighini and Harris, 2008). Further experiments performed in *M. grisea* showed that the inhibition or scavenging of ROS production by the NADPH oxidase inhibitor DPI or by the antioxidant ascorbate inhibited or impaired fungal polarized growth, which was detected as inhibition or delay of germination of the conidia and aberrant morphology of the germ tubes or appressoria (Egan et al., 2007). In the mutualistic, endophytic fungus *E. festucae*, it was demonstrated that ROS generation requires the functional assembly of a multisubunit complex composed of NoxA, a regulatory component, NoxR, and the small GTPase RacA (Takemoto et al., 2007; Tanaka et al., 2008), whereas BemA and Cdc24, well-characterized regulators of polarity in yeast, were identified as interacting with the Nox complex via NoxR (Takemoto et al., 2011). Significantly, GFP fusions of NoxR, RacA, Cdc24, and BemA preferentially localized to actively growing hyphal tips, where they functioned as an activated NADPH oxidase enzyme complex responsible for the production of ROS (Takemoto et al., 2011). These results together with our observations indicate that the NADPH oxidase-dependent production of ROS plays a conserved role in polarized hyphal growth. It is well known that the fungal cytoskeleton plays a crucial role in polarity establishment, maintenance and polar growth (Harris, 2006; Riquelme, 2013). This finding was validated in *Pst* by functional analysis of the actin gene *PsACT1* (Liu et al., 2012). A recent study revealed that ROS may regulate filamentous polarized fungal growth by remodeling the arrangement of the F-actin cytoskeleton, whereas the latrunculin-mediated depolymerization of fungal appressorial F-actin is competitively inhibited by fungal NADPH oxidases mediated by ROS (Rydera et al., 2013). Thus, we can speculate that the production of ROS by the *Pst* NADPH oxidase complex in *Pst* regulates polarized growth by reorganizing components of the cytoskeleton, such as F-actin.

In addition to ROS, we provide evidence that NO is involved in polarized growth during spore germination and subsequent germ tube growth. As with ROS, interference in NO production by application of scavenger or promoter delayed spore germination and impaired germ tube growth. A similar role has also been indicated in other fungi. For instance, the application of external NO to *C. coccodes* delayed spore germination, whereas treatment with L-Arg accelerated the germination and development of conidiospores (Wang and Higgins, 2005). Similarly, in the hemibiotrophic ascomycete *M. oryzae*, NO scavengers delayed germination and reduced lesion formation (Samalova et al., 2013). These data and our observations collectively indicate that NO may have a role in signaling in spore germination and polarized growth in fungi.

Significantly, NO seems to act in concert with ROS to control germination and germ tube growth because the elevation or reduction of NO or ROS alone has a negative effect on these processes, whereas the accumulation of high level of NO and ROS results in normal growth in *Pst.* It is likely that a balance between NO and ROS, rather than these molecules functioning alone, allows germination to proceed while ensuring that it does so only under ideal environmental conditions (Wang and Higgins, 2005).

It has been demonstrated that NO and ROS signaling pathways in plant biotic interactions are closely connected (Scheler et al., 2013). Furthermore, there is evidence showing that ROS can influence NO levels and vice versa (Moncada and Erusalimsky, 2002; Desikan et al., 2004). For example, the regulation of ROS production by NO is thought to modulate the development of the hypersensitive response (HR), a programmed cell death involved in plant defense (Yun et al., 2011; Rasul et al., 2012). NO and ROS crosstalk during fungal development may be resolved only after the characterization of all fungal NOS isomers (Wang and Higgins, 2005). We found that upon treatment with either the promoter or scavenger of NO, ROS generation in *Pst* urediniospores was inhibited. Similarly, upon treatment with either the donor or scavenger of ROS, the generation of NO was inhibited. A critical balance of ROS and NO seems to be essential in regulating urediniospore germination and germ tube development in *Pst* and other fungi.

The generation of ROS during the interaction of fungus and its host has been repeatedly studied. There is evidence that the host produces ROS (Wang et al., 2007, 2010; Zhang et al., 2012) and NO (Romero-Puertas et al., 2004; Piterkova et al., 2009; Sedlářvá et al., 2010; Melillo et al., 2011) during the early stages of infection. In incompatible interactions, ROS was detected in the stomata and the necrotic mesophyll cells following fungal penetration and the induction of HR (Wang et al., 2007, 2010). Similar results were obtained by our laboratory with regard to NO (Yin et al., unpublished). The generation of ROS and NO is also involved in systemic acquired resistance (Gao et al., 2014; Wendehenne et al., 2014).

Combining all of the results, we deduced that in addition to supplying itself for apical growth, it was likely that *Pst* also produced a small amount of ROS and NO to adapt to the highly oxidative conditions in the infected plant. The small amount of ROS and NO might protect the growth and development of the germ tube and the expansion of hyphae in the intercellular space from the harm of highly oxidative condition in the infected plant.

In future studies, a higher priority should be given to defining the molecular identity of the genes involved in NO and ROS biosynthesis and the relationship between NO and ROS in signaling transduction during germination and germ tube growth in *Pst*.

## Author Contributions

ZK and HZ designed experiments; SY carried out experiments; ZG analyzed experimental results; CW and LH joined the discussion and gave the original ideas; SY wrote the manuscript.

## Conflict of Interest Statement

The authors declare that the research was conducted in the absence of any commercial or financial relationships that could be construed as a potential conflict of interest. The reviewer JA and handling Editor declared a current collaboration and the handling Editor states that the process nevertheless met the standards of a fair and objective review.

## Abbreviations

CLSM: confocal laser scanning microscopy
c-PTIO: 2-(4-carboxyphenyl)-4,4,5,5-tetramethylimidazoline-1-oxyl-3-oxide
DAF-FM DA: 4-amino-5-(*N*-methylamino)-2, 7-difluorofluorescein diacetate
H_2_DCF DA: 2′, 7′-dichlorodihydrofluorescein diacetate
DAF-FM T: 4-amino-5-(*N*-methylamino)-2, 7-difluorofluorescein triazole
DCF: dichlorofluorescein
DPI: diphenyliodonium iodide
HPG: hours post germination
L-NAME: *N*-nitro-L-arginine methyl ester
MFI: mean fluorescence intensity
MGL: mean germ tube lengths
MGR: mean germination rate
NADPH: nicotinamide adenine dinucleotide phosphate
NBT: nitroblue tetrazolium
NO: nitric oxide
NOS: nitric oxide synthase
*Pst*: *Puccinia striiformis* f. sp. *Tritici;*
RNS: reactive nitrogen species
ROS: reactive oxygen species

## References

[B1] Averyanov A. A. Lapikova V. P. (1990). Activated oxygen as a possible factor in the autoinhibition of spore germination of the fungus *Pyricularia oryzae*. *Biochemistry* 55 1397–1402.

[B2] Balcerczyk A. Soszynski M. Bartosz G. (2005). On the specificity of 4-amino-5-methylamino-2’,7’ -difluorofluorescein as a probe for nitric oxide. *Free Radical Biol. Med.* 39 327–335. 10.1016/j.freeradbiomed.2005.03.017 15993331

[B3] Besson-Bard A. Pugin A. Wendehenne D. (2008). New insights into nitric oxide signialling in plants. *Annu. Rev. Plant Biol.* 59 21–39. 10.1146/annurev.arplant.59.032607.092830 18031216

[B4] Bonilla I. El-Hamdaoui A. Bolaños L. (2004). Boron and calcium increase *Pisum sativum* seed germination and seedling development under salt stress. *Plant Soil* 267 97–107. 10.1007/s11104-005-4689-7

[B5] Brisson L. F. Tenhaken R. Lamb C. (1994). Function of oxidative cross-linking of cell wall structural proteins in plant disease resistance. *Plant Cell* 6 1703–1712. 10.2307/3869902 12244231 PMC160556

[B6] Castello P. R. Woo D. K. Ball K. Wojcik J. Liu L. Poyton R. O. (2008). Oxygen-regulated isoforms of cytochrome c oxidase have differential effects on its nitric oxide production and on hypoxic signaling. *Proc. Natl. Acad. Sci. U.S.A.* 105 8203–8208. 10.1073/pnas.0709461105 18388202 PMC2448815

[B7] Chaki M. Fernandez-Ocana A. M. Valderrama R. Carreras A. Esteban F. J. Luque F. (2009). Involvement of reactive nitrogen and oxygen species (RNS and ROS) in sunflower-mildew interaction. *Plant Cell Physiol.* 50 265–279. 10.1093/pcp/pcn196 19112080

[B8] Chen W. Q. Wellings C. Chen X. M. Kang Z. S. Liu T. G. (2014). Wheat stripe (yellow) rust caused by *Puccinia striiformis* f. sp. tritici. *Mol. Plant Pathol.* 15 433–446. 10.1111/mpp.12116 24373199 PMC6638732

[B9] Chen X. M. (2005). Epidemiology and control of stripe rust [*Puccinia striiformis* f. sp. tritici] on wheat. *Can. J. Plant Pathol.* 27 314–337. 10.1080/07060660509507230

[B10] Delledonne M. Xia Y. J. Dixon R. A. Lamb C. (1998). Nitric oxide functions as a signal in plant disease resistance. *Nature* 394 585–588. 10.1038/29087 9707120

[B11] Desikan R. Cheung M. K. Bright J. Henson D. Hancock J. T. Neill S. J. (2004). ABA, hydrogen peroxide and nitric oxide signalling in stomatal guard cells. *J. Exp. Bot.* 55 205–212. 10.1093/jxb/erh033 14673026

[B12] Egan M. J. Wang Z. Y. Jones M. A. Smirnoff N. Talbot N. J. (2007). Generation of reactive oxygen species by fungal NADPH oxidases is required for rice blast disease. *Proc. Natl. Acad. Sci. U.S.A.* 104 11772–11777. 10.1073/pnas.0700574104 17600089 PMC1913907

[B13] Elkins J. G. Hassett D. J. Stewart P. S. Schweizer H. P. McDermott T. R. (1999). Protective role of catalase in *Pseudomonas aeruginosa* biofilm resistance to hydrogen peroxide. *Appl. Environ. Microbiol.* 65 4594–4600. 10508094 10.1128/aem.65.10.4594-4600.1999 PMC91612

[B14] Finkel T. (2003). Oxidant signals and oxidative stress. *Curr. Opin. Cell Biol.* 15 247–254. 10.1016/S0955-0674(03)00002-4 12648682

[B15] Foreman J. Demidchik V. Bothwell J. H. Mylona P. Miedema H. Torres M. A. (2003). Reactive oxygen species produced by NADPH oxidase regulate plant cell growth. *Nature* 422 442–446. 10.1038/nature01485 12660786

[B16] Gao Q. Yu K. Xia Y. Shine M. B. Wang C. Navarre D. (2014). Mono- and digalactosyldiacylglycerol lipids function nonredundantly to regulate systemic acquired resistance in plants. *Plant Cell Rep.* 9 1681–1691. 10.1016/j.celrep.2014.10.069 25466253

[B17] Glasauer A. Chandel N. S. (2013). ROS. *Curr. Biol.* 23 100–102. 10.1016/j.cub.2012.12.011 23391379

[B18] Gong X. Fu Y. Jiang D. Li G. Yi X. Peng Y. (2007). L-arginine is essential for conidiation in the filamentous fungus *Coniothyrium minitans*. *Fungal Genet. Biol.* 44 1368–1379. 10.1016/j.fgb.2007.07.007 17897846

[B19] Harris S. D. (2006). Cell polarity in filamentous fungi: shaping the mold. *Int. Rev. Cytol.* 251 41–77. 10.1016/S0074-7696(06)51002-2 16939777

[B20] Heller J. Tudzynski P. (2011). Reactive oxygen species in phytopathogenic fungi: signaling, development and disease. *Annu. Rev. Phytopathol.* 49 369–390. 10.1146/annurev-phyto-072910-095355 21568704

[B21] Kwak J. M. Mori I. C. Pei Z. M. Leonhardt N. Torres M. A. Dangl J. L. (2003). NADPH oxidase AtrbohD and AtrbohF genes function in ROS-dependent ABA signaling in *Arabidopsis*. *EMBO J.* 22 2623–2633. 10.1093/emboj/cdg277 12773379 PMC156772

[B22] Lambeth J. D. (2004). NOX enzymes and the biology of reactive oxygen. *Nat. Rev. Immunol.* 4 181–189. 10.1038/nri1312 15039755

[B23] Levine A. Tenhaken R. Dixon R. Lamb C. (1994). H_2_O_2_ from the oxidative burst orchestrates the plant hypersensitive disease resistance response. *Cell* 79 583–593. 10.1016/0092-8674(94)90544-4 7954825

[B24] Liu J. Zhang Q. Chang Q. Zhuang H. Huang L. L. Kang Z. S. (2012). Cloning and characterization of the actin gene from *Puccinia striiformis* f. sp. tritici. *World J. Microbiol. Biotechnol.* 28 2331–2339. 10.1007/s11274-012-1040-3 22806107

[B25] Lu H. Higginsf V. J. (1999). The effect of hydrogen peroxide on the viability of tomato cells and of the fungal pathogen *Cladosporium fulvum*. *Physiol. Mol. Plant Pathol.* 54 131–143. 10.1006/pmpp.1998.0195

[B26] Maier J. Hecker R. Rockel P. Ninnemann H. (2001). Role of nitric oxide synthase in the light-induced development of sporangiophores in *Phycomyces blakesleeanus*. *Plant Physiol.* 126 1323–1330. 10.1104/pp.126.3.1323 11457983 PMC116489

[B27] Melillo M. T. Leonetti P. Leone A. Veronico P. Bleve-Zacheo T. (2011). ROS and NO production in compatible and incompatible tomato-Meloidogyne incognita interactions. *Eur. Plant Pathol.* 130 489–502. 10.1007/s10658-011-9768-4

[B28] Michan S. Lledias F. Baldwin J. D. Natvig D. O. Hansberg W. (2002). Regulation and oxidation of two large monofunctional catalases. *Free Radical Biol. Med.* 33 521–532. 10.1016/S0891-5849(02)00909-7 12160934

[B29] Momany M. (2002). Polarity in filamentous fungi: establishment, maintenance and new axes. *Curr. Opin. Microbiol.* 5 580–585. 10.1016/S1369-5274(02)00368-5 12457701

[B30] Moncada S. Erusalimsky J. D. (2002). Does nitric oxide modulate mitochondrial energy generation and apoptosis. *Nat. Rev. Mol. Cell Biol.* 3 214–220. 10.1038/nrm762 11994742

[B31] Ninnemann H. Maier J. (1996). Indications for the occurrence of nitric oxide synthases in fungi and plants and the involvement in photoconidiation of *Neurospora crassa*. *Photochem. Photobiol.* 64 393–398. 10.1111/j.1751-1097.1996.tb02477.x 8760579

[B32] Nowles R. G. Moncada S. (1994). Nitric oxide synthases in mammals. *Biochem. J.* 298 249–258. 10.1042/bj2980249 7510950 PMC1137932

[B33] Palmer R. M. J. Ashton D. S. Moncada S. (1987). Vascular endothelial cells synthesize nitric oxide from L-arginine. *Nature* 333 664–666. 10.1038/333664a0 3131684

[B34] Piterkova J. Petrivalsky M. Luhova L. Mieslerova B. Sedlarova M. Lebeda A. (2009). Local and systemic production of nitric oxide in tomato responses to powdery mildew infection. *Mol. Plant Pathol.* 10 501–513. 10.1111/j.1364-3703.2009.00551.x 19523103 PMC6640527

[B35] Prats E. Carver T. L. Mur L. A. (2008). Pathogen-derived nitric oxide influences formation of the appressorium infection structure in the phytopathogenic fungus *Blumeria graminis*. *Res. Microbiol.* 159 476–480. 10.1016/j.resmic.2008.04.001 18554873

[B36] Rasul S. Dubreuil-Maurizi C. Lamotte O. Koen E. Poinssot B. Alcaraz G. (2012). Nitric oxide production mediates oligogalacturonide-triggered immunity and resistance to *Botrytis cinerea* in *Arabidopsis thaliana*. *Plant Cell Environ.* 35 1483–1499. 10.1111/j.1365-3040.2012.02505.x 22394204

[B37] Riquelme M. (2013). Tip growth in filamentous fungi: a road trip to the apex. *Annu. Rev. Microbiol.* 67 587–609. 10.1146/annurev-micro-092412-155652 23808332

[B38] Romero-Puertas M. C. Perazzolli M. Zago E. D. Delledonne M. (2004). Nitric oxide signalling functions in plant-pathogen interactions. *Cell Microbiol.* 6 795–803. 10.1111/j.1462-5822.2004.00428.x 15272861

[B39] Rydera L. S. Dagdasa Y. F. Mentlaka T. A. Kershawa M. J. Thorntona C. R. Schustera M. (2013). NADPH oxidases regulate septin-mediated cytoskeletal remodeling during plant infection by the rice blast fungus. *Proc. Natl. Acad. Sci. U.S.A.* 110 3179–3184. 10.1073/pnas.1217470110 23382235 PMC3581893

[B40] Samalova M. Johnson J. Illes M. Kelly S. Fricker M. Gurr S. (2013). Nitric oxide generated by the rice blast fungus *Magnaporthe oryzae* drives plant infection. *New Phytol.* 197 207–222. 10.1111/j.1469-8137.2012.04368.x 23072575

[B41] Scheler C. Durner J. Astier J. (2013). Nitric oxide and reactive oxygen species in plant biotic interactions. *Curr. Opin. Plant Biol.* 16 534–539. 10.1016/j.pbi.2013.06.020 23880111

[B42] Sedlářvá M. Petřivalský M. Piterková J. Luhová L. Kočířová J. Lebeda A. (2010). Influence of nitric oxide and reactive oxygen species on development of lettuce downy mildew in *Lactuca* spp. *Eur. Plant Pathol.* 129 267–280. 10.1007/s10658-010-9626-9

[B43] Semighini C. P. Harris S. D. (2008). Regulation of apical dominance in aspergillus nidulans hyphae by reactive oxygen species. *Genetics* 179 1919–1932. 10.1534/genetics.108.089318 18689883 PMC2516069

[B44] Swanson S. Gilroy S. (2010). ROS in plant development. *Physiol. Plant.* 138 384–392. 10.1111/j.1399-3054.2009.01313.x 19947976

[B45] Takemoto D. Kamakura S. Saikia S. Becker Y. Wrenn R. Tanaka A. (2011). Polarity proteins Bem1 and Cdc24 are components of the filamentous fungal NADPH oxidase complex. *Proc. Natl. Acad. Sci. U.S.A.* 108 2861–2866. 10.1073/pnas.1017309108 21282602 PMC3041104

[B46] Takemoto D. Tanaka A. Scott B. (2007). NADPH oxidases in fungi: diverse roles of reactive oxygen species in fungal cellular differentiation. *Fungal Genet. Biol.* 44 1065–1076. 10.1016/j.fgb.2007.04.011 17560148

[B47] Tanaka A. Christensen M. J. Takemoto D. Park P. Scotta B. (2006). Reactive oxygen species play a role in regulating a fungus–perennial ryegrass mutualistic interaction. *Plant Cell* 18 1052–1066. 10.1105/tpc.105.039263 16517760 PMC1425850

[B48] Tanaka A. Takemoto D. Hyon G. S. Park P. Scott B. (2008). NoxA activation by the small GTPase RacA is required to maintain a mutualistic symbiotic association between Epichloë festucae and perennial ryegrass. *Mol. Microbiol.* 68 1165–1178. 10.1111/j.1365-2958.2008.06217.x 18399936

[B49] Turrion-Gomez J. L. Benito E. P. (2011). Flux of nitric oxide between the necrotrophic pathogen *Botrytis cinerea* and the host plant. *Mol. Plant Pathol.* 12 606–616. 10.1111/j.1364-3703.2010.00695.x 21722298 PMC6640425

[B50] Vieira A. L. Linares E. Augusto O. Gomes S. L. (2009). Evidence of a Ca^2+^-NO-cGMP signaling pathway controlling zoospore biogenesis in the aquatic fungus *Blastocladiella emersonii*. *Fungal Genet. Biol.* 46 575–584. 10.1016/j.fgb.2009.04.002 19393757

[B51] Wang C. F. Huang L. L. Buchenauer H. Han Q. M. Zhang H. C. Kang Z. S. (2007). Histochemical studies on the accumulation of reactive oxygen species (O_2_- and H_2_O_2_) in the incompatible and compatible interaction of wheat - *Puccinia striiformis* f. sp tritici. *Physiol. Mol. Plant Pathol.* 71 230–239. 10.1016/j.pmpp.2008.02.006

[B52] Wang C. F. Huang L. L. Zhang H. C. Han Q. M. Buchenauer H. Kang Z. S. (2010). Cytochemical localization of reactive oxygen species (O_2_- and H_2_O_2_) and peroxidase in the incompatible and compatible interaction of wheat-*Puccinia striiformis* f. sp. tritici. *Physiol. Mol. Plant Pathol.* 74 221–229. 10.1016/j.pmpp.2010.02.002

[B53] Wang J. Higgins V. J. (2005). Nitric oxide has a regulatory effect in the germination of conidia of *Colletotrichum coccodes*. *Fungal Genet. Biol.* 42 284–292. 10.1016/j.fgb.2004.12.006 15749048

[B54] Wendehenne D. Gao Q. M. Kachroo A. Kachroo P. (2014). Free radical-mediated systemic immunity in plants. *Curr. Opin. Plant Biol.* 20 127–134. 10.1016/j.pbi.2014.05.012 24929297

[B55] Wilson I. D. Neill S. J. Hancock J. T. (2008). Nitric oxide synthesis and signalling in plants. *Plant Cell Environ.* 31 622–631. 10.1111/j.1365-3040.2007.01761.x 18034772

[B56] Yun B. Feechan A. Yin M. Saidi N. Le Bihan T. Yu M. (2011). S-nitrosylation of NADPH oxidase regulates cell death in plant immunity. *Nature* 478 264–268. 10.1038/nature10427 21964330

[B57] Zhang H. C. Wang C. F. Cheng Y. L. Chen X. M. Han Q. M. Huang L. L. (2012). Histological and cytological characterization of adult plant resistance to wheat stripe rust. *Plant Cell Rep.* 31 2121–2137. 10.1007/s00299-012-1322-0 22833277

[B58] Zheng W. Huang L. Huang J. Wang X. Chen X. Zhao J. (2013). High genome heterozygosity and endemic genetic recombination in the wheat stripe rust fungus. *Nat. Commun.* 4:2673 10.1038/ncomms3673 PMC3826619 24150273

